# Behavioral Characteristics of Medical Students on Social Media Platforms: Topic Modeling and Social Network Analysis

**DOI:** 10.2196/87304

**Published:** 2026-08-07

**Authors:** Yongjie Li, Yangshan Fu, Fenshuang Zheng, Xiangyu Yan

**Affiliations:** 1 Health Science Center, Peking University, Beijing, Beijing, China; 2 The Affiliated Hospital of Yunnan University, No. 176 Qingnian Road, Kunming, Yunnan, China, 86 0871-63627114; 3 School of Disaster and Emergency Medicine, Tianjin University, Tianjin, Tianjin, China

**Keywords:** medical education, social network analysis, topic modeling, online communities, medical students

## Abstract

**Background:**

Medical students experience sustained academic, clinical, and psychosocial pressures. Online forums provide an informal space where students seek information, express concerns, and exchange peer support. However, few studies have analyzed these interactions longitudinally based on real-world data from online social platforms.

**Objective:**

This study aimed to integrate topic modeling, temporal topic analysis, and social network analysis (SNA) to investigate evolving concerns, network positions, and influence structures within the bulletin board system of a medical university over a 7-year period.

**Methods:**

We collected all posts and comments from the “Medical Center” board of the Peking University bulletin board system forum between 2017 and 2023. After excluding irrelevant posts, nontextual elements, blank content, and noise terms, the textual corpus was segmented and standardized for analysis. Latent Dirichlet allocation (LDA) was used for topic modeling to identify primary discussion topics. The number of topics was selected through 5-fold cross-validation by evaluating candidate models with 5 to 20 topics using perplexity. Annual topic prevalence was calculated to examine temporal changes in student concerns. SNA was also used to construct interaction networks. Users were defined as nodes, and directed edges were established when one user replied to another user’s post or comment. Degree centrality, eigenvector centrality, and betweenness centrality were calculated to identify active users, influential hubs, and bridging users. A topic-network coupling analysis further linked topic categories with the centrality measures of users.

**Results:**

The corpus included 4639 posts and 58,942 comments. The annual post numbers fluctuated over the years, reaching their lowest point in 2019 and peaking in 2022. Posting activity increased after 8 AM, remained relatively stable from noon to 10 PM, and declined after 10 PM. Seven major topics of concern were identified, with feedback on living facilities (1025/4639, 22.1%) being the most prevalent, followed by information inquiry (1004/4639, 21.6%) and medical education (883/4639, 19.0%). Temporal analysis showed that concerns related to living facilities remained a recurring topic across years, and emotional support remained a low-frequency but persistent topic. The overall network was sparse, with a small subset of users occupying active, influential, or bridging positions. Topic-network coupling showed that high-volume topics were not necessarily network-central: feedback on living facilities had the largest discussion volume but the lowest median centrality values, whereas current affairs had the highest median degree and eigenvector centrality, and psychological and emotional support had the highest median betweenness centrality.

**Conclusions:**

Online forums can reveal not only what students discuss but also how concerns persist, change, and circulate through peer interaction networks. Combining topic modeling with network analysis may help educational administrators identify persistent concerns related to the learning environment, time-sensitive issues, and structurally important topics requiring targeted communication or support strategies.

## Introduction

Medical education is widely recognized as a period of intense academic and personal challenges [1]. Compared with the general student population, medical students face a demanding curriculum, a protracted training cycle, and significant emotional pressures, factors that collectively contribute to high rates of stress, burnout, and mental health issues in this population [2,3]. Beyond the formal curriculum, students’ overall well-being, sense of belonging, and academic success are profoundly influenced by their learning and living environment [4]. Factors such as campus facilities and support services are critical components that shape the student experience, potentially mitigating or exacerbating the stresses inherent in medical training. In this context, peer support networks serve as a vital resource, providing academic help, emotional support, and a sense of community that is crucial for navigating the high demands of medical school [5].

In recent years, the development of digital technology has reshaped how students interact and communicate. Online platforms, such as bulletin board system (BBS) forums and social media, have become central hubs for student exchange [6]. These digital spaces offer accessible arenas for information seeking, peer support, and informal learning outside the classroom [7,8]. The importance of these online communities was further amplified during global crises such as the COVID-19 pandemic, which shifted many aspects of student life online and highlighted the critical role of digital platforms in maintaining social connections and disseminating vital information [9,10].

Previous studies have shown that understanding students’ needs relies on methods such as surveys and focus groups [11-13]. While valuable, these approaches can be constrained by recall bias and may fail to capture the spontaneous, real-time concerns of students. The rich data available in online forums present an opportunity to overcome these limitations.

The rapid advancement of machine learning has introduced a new paradigm for educational research. Topic modeling and social network analysis (SNA) provide powerful tools for unlocking insights from complex digital data. Topic modeling techniques, such as latent Dirichlet allocation (LDA), can automatically identify latent themes within large text corpora, revealing the primary topics of discussion [14]. Meanwhile, SNA offers a methodology to map and quantify social interactions, uncovering behavioral patterns, information diffusion pathways, and the overall community structure [15-18]. Despite the promise of these methods, there is a scarcity of research that combines both topic modeling and SNA to examine medical students’ online behaviors. Furthermore, longitudinal analyses tracking how these topics and influence structures evolve over time remain limited.

Therefore, this study uses a combination of topic modeling and SNA to conduct a 7-year (2017‐2023) longitudinal investigation of medical students’ BBS activity. The research aims to identify the core topics of concerns and their prevalence over time, map the community’s social network structure, identify key influential users, and analyze the dynamic evolution of the network structure. The findings are intended to provide educational administrators and student support services with data-driven insights to enhance support strategies and inform campus governance in medical education.

## Methods

### Data Source

The dataset was collected from the “Medical Center” board of a BBS forum, encompassing all posts published between 2017 and 2023, including post titles, content, time stamps, user IDs, and other relevant metadata. The BBS forum of Peking University, officially launched in 2000, is a campus-wide system primarily serving the university’s network [19]. It has attracted widespread participation from students and faculty.

To ensure analytical accuracy and validity, raw unstructured data were subjected to cleaning and preprocessing. Initial filtering removed post types not relevant to the research objectives, such as advertisements, recruitment notices, administrative announcements, and forum bulletins. As this study was designed as a text-based analysis, nontextual elements (images and videos), blank content, and nonessential tokens (emojis, punctuation, and HTML tags) were then removed. High-frequency noise terms were identified via term-frequency screening and, together with a stop-word list, were excluded to improve corpus quality. Finally, the Jieba toolkit was used for word segmentation, enabling further cleaning and standardization of the text to construct the corpus [20]. All user identifiers were anonymized to comply with ethical standards and privacy protection requirements.

### Text Analytics

A multidimensional text analysis framework was used to examine behavioral patterns and topics of interest among medical students on the platform. Annual post counts were aggregated and visualized as line charts to illustrate longitudinal trends, while diurnal posting frequencies were analyzed to reflect daily activity patterns. Topic modeling was performed using LDA, an unsupervised machine learning method widely used to extract latent themes from large-scale textual data.

### Model Selection and Topic Validation

The number of topics was treated as the primary hyperparameter for the LDA model. Candidate models with topic numbers ranging from 5 to 20 were evaluated using 5-fold cross-validation, and model performance was assessed by perplexity, with lower values indicating better fit [21]. Following model training, keywords representative of each topic (10 keywords) were analyzed semantically and visualized to aid interpretation. To improve interpretability, the top 10 keywords and representative texts for each of the 16 topics were reviewed by the research team. Semantically overlapping topics were consolidated into broader categories.

### Temporal Topic Analysis

To examine changes in student concerns over time, we calculated the annual prevalence of each topic category from 2017 to 2023. For each year, the number of posts assigned to each topic was divided by the total number of topic-assigned posts in that year. The resulting annual topic proportions were visualized to identify stable, increasing, or declining patterns in student concerns over the study period.

### Social Network Construction and Analysis

We applied SNA to interactions on the BBS platform to characterize the overall community structure, interaction frequencies, and information diffusion patterns [22,23]. Social networks were constructed using the NetworkX library and visualized with the Matplotlib library in Python (Python Software Foundation) to examine social connectivity and the influence of core nodes.

### Network Definition and Construction

Individual users were defined as nodes, and reply-based interactions between users were defined as edges. A directed edge was established from user A to user B if A replied to a post or comment by B. Node size was proportional to interaction frequency. To capture temporal evolution, annual networks were constructed from 2017 to 2023, enabling dynamic analysis of structural changes over time.

### Core Metric Analysis

To probe network structure, we computed three standard centralities:

1. Degree centrality: measures the number of direct connections per node, reflecting user activity
2. Eigenvector centrality: assesses a node’s global influence based on connections to other well-connected nodes, identifying pivotal actors in information flow
3. Betweenness centrality: quantifies a node’s role as a bridge in information pathways, highlighting its capacity to connect disparate subgroups [24].

### Topic-Network Coupling Analysis

To examine whether different discussion topics occupied different positions within the interaction network, we linked each consolidated topic category with user-level centrality measures. For each topic, we calculated the number of posts, number of comments, number of participating users, and the median degree centrality, eigenvector centrality, and betweenness centrality of participating users. This analysis was used to examine whether some topics were more central or peripheral within the forum’s interaction structure.

### Visualization and Dynamic Network Evolution

A heatmap was used to visualize the annual top 10 nodes across the 3 centrality metrics, with darker colors indicating higher values. As core nodes varied across years, the total number of nodes in the heatmap exceeded 10. Subnetworks containing these core nodes were extracted annually, with core nodes and their direct ties highlighted in red to illustrate their central positioning and radial influence. This approach facilitated the observation of evolving core-periphery structures and information propagation mechanisms.

### Ethical Considerations

This study was reviewed and approved by the Ethics Committee of Tianjin University (TJUE2026-H-S-103). The requirement for informed consent was waived by the ethics committee because this study involved retrospective analysis of anonymized online forum data. All user identifiers were anonymized before analysis, and no personally identifiable information was reported.

## Results

**Figure 1.**
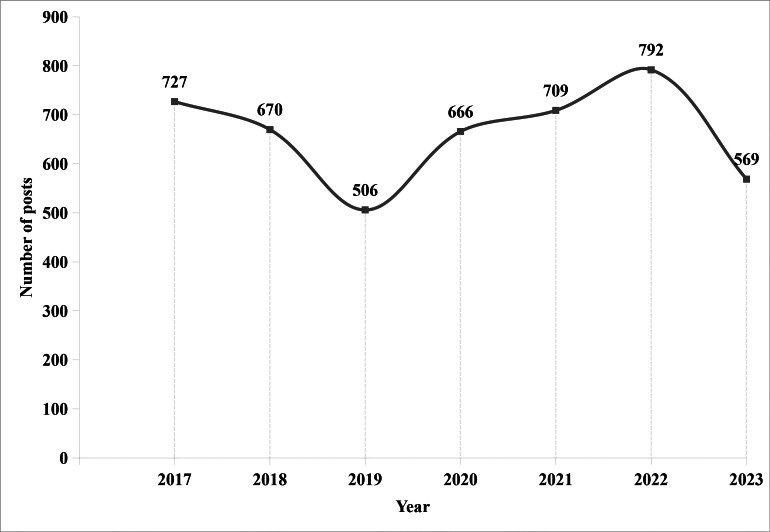
Annual number of user posts.

**Figure 2.**
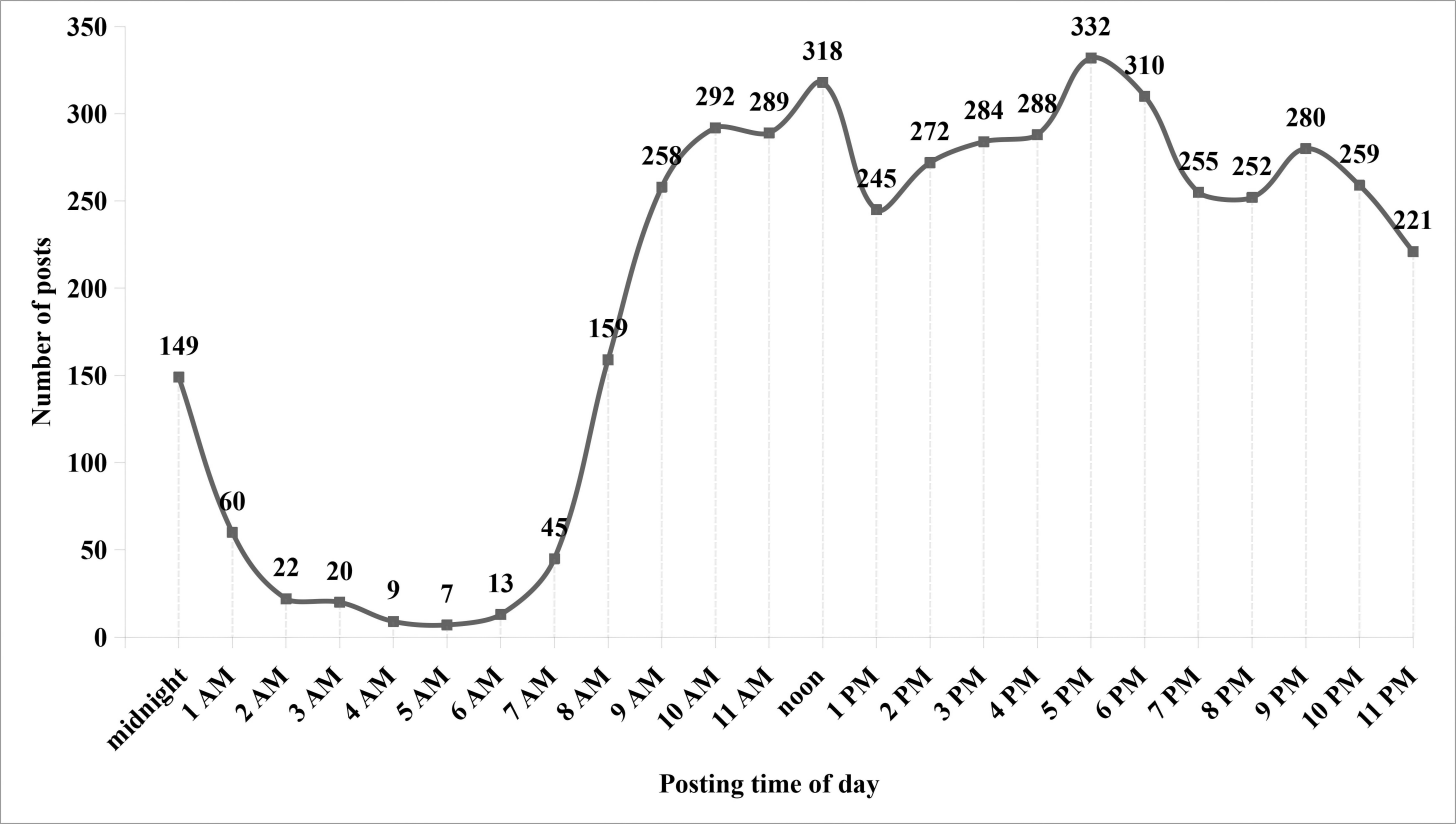
User posting frequency.

By December 31, 2023, a total of 4639 posts and 58,942 comments published from 2017 to 2023 were included for statistical analysis after data preprocessing. The annual number of posts showed a fluctuating trend over the study period: after reaching a low in 2019, post counts gradually recovered and peaked in 2022, followed by a decline in 2023 (Figure 1). Diurnal activity patterns revealed clear temporal fluctuations in posting frequency. Posting activity began to increase from 8 AM, reached higher levels after 10 AM, remained relatively stable throughout the afternoon and evening (noon to 10 PM), and gradually declined after 10 PM (Figure 2). These patterns reflect consistent daily behavioral rhythms, providing data-driven insights into the lifestyles and social habits of medical students. To identify central themes in user-generated content, topic modeling was performed using LDA. During the 5-fold cross-validation, topic models with numbers ranging from 5 to 20 were evaluated. The optimal number of topics was determined to be 16. After consolidation of semantically overlapping themes, 7 major topics were identified: feedback on living facilities, information inquiry, medical education, feedback on campus services, campus policies, current affairs, and psychological and emotional support. Table 1 presents representative keywords and the prevalence of each topic.

**Table 1.** Topic classification and keywords in the bulletin board system corpora.

Classification and topics	Keywords	Proportion, n (%)
Feedback on living facilities	Library, canteen, Yi-Fu building, renovation, registration, swimming pool, construction, curfew, and noise	1025 (22.1)
Information inquiry	Treatment or therapy, surgery, reimbursement, expert, public medical services, seeking help, postgraduate entrance exam, alumni, and paid service	1004 (21.6)
Medical education	Clinical, training, thesis or dissertation, career path, curriculum vitae, internship, graduation, postdoctoral research, and program	883 (19.0)
Feedback on campus services	Taste, dishes, charging, printing, renovation, temperature, fruits, suggestion, and campus network	865 (18.6)
Campus policies	Main campus, access, registration, security office, scholarship, early notice, notification, shuttle bus, and opening hours	565 (12.2)
Current affairs	Virus, journalist, Haidian District, everyone, news, public opinion, vaccine, vaccination, and artificial intelligence	195 (4.2)
Psychological and emotional support	Curious, annoyed, disturbed sleep, seeking help, emotional expression, communication, cooperation, mentor, and classmates	102 (2.2)

Feedback on living facilities (1025/4639, 22.1%) was the most frequent topic, covering facilities closely related to student life such as supermarkets, dormitories, canteens, libraries, and gyms, reflecting a strong demand for improved living conditions and campus experience. Information inquiry (1004/4639, 21.6%) indicated high reliance on academic, examination-related, and career development information, underscoring the need for accurate and efficient information services. Medical education (883/4639, 19.0%) captured concerns about professional training, academic advancement, and career planning, highlighting students’ focus on personal growth. Feedback on campus services (865/4639, 18.6%) conveyed ongoing attention to service quality, including medical care and transportation, suggesting a need for optimized resource allocation. Although psychological and emotional support accounted for a relatively small proportion of total posts, it revealed underlying needs for mental stress relief and emotional support, indicating the importance of enhanced psychological education and intervention. The current affairs topic reflected students’ awareness of and engagement with major social events, demonstrating a sense of social responsibility.

**Figure 3.**
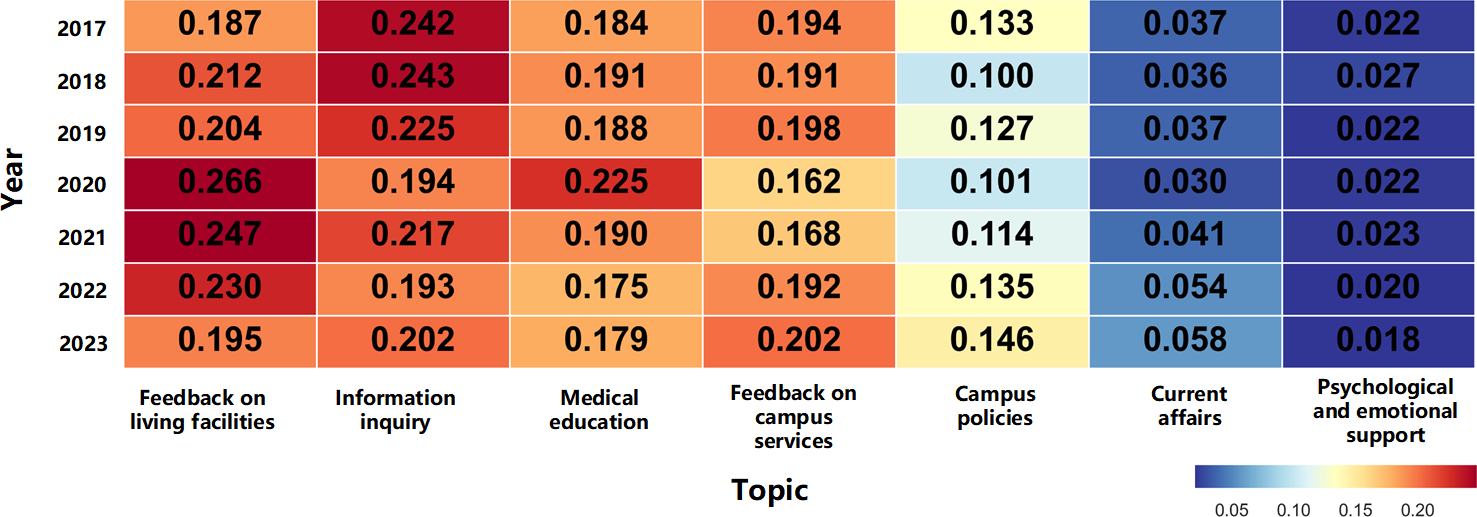
Annual prevalence of topic categories from 2017 to 2023.

To further examine whether student concerns changed over time, we analyzed the annual prevalence of the 7 topic categories from 2017 to 2023 (Figure 3). Feedback on living facilities remained the most prominent topic across most years (136/727, 18.7% to 177/666, 26.6%). Information inquiry declined from 24.2% (176/727) in 2017 to 19.4% (129/666) in 2020 and 19.3% (153/792) in 2022, before increasing slightly to 20.2% (115/569) in 2023. Medical education remained relatively stable over time, ranging from 17.5% (139/792) to 22.5% (150/666) across the study period. Psychological and emotional support remained a low-frequency but persistent topic, ranging from 1.8% (10/569) to 2.3% (16/709) across years. Current affairs showed a gradual increase in later years, rising from 3.0% (20/666) in 2020 to 5.8% (33/569) in 2023.

**Figure 4.**
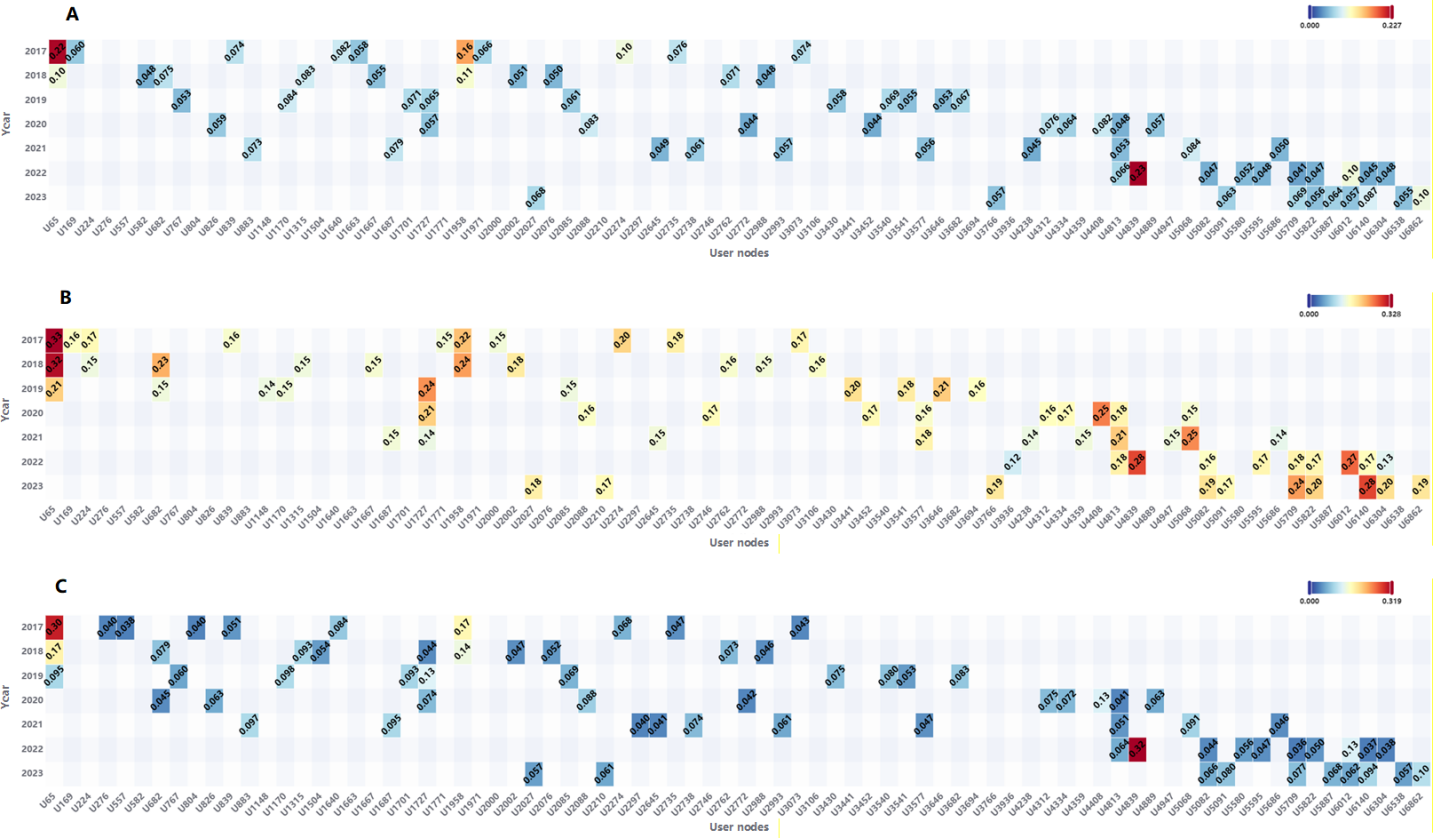
Heatmap of network metrics for core nodes. (A) Heatmap of degree centrality (top 10 nodes), (B) heatmap of eigenvector centrality (top 10 nodes), and (C) heatmap of betweenness centrality (top 10 nodes).

Heatmaps clearly illustrated the distribution and evolution of influential nodes within the social network, highlighting the key roles of nodes with high centrality values in facilitating information diffusion (Figure 4). The overall analysis of the network from 2017 to 2023 showed generally low centrality values. Specifically, the median degree centrality was 0.000745 (IQR 0.000521‐0.001815), the median eigenvector centrality was 0.004527 (IQR 0.001946‐0.009820), and the median betweenness centrality was 0.000011 (IQR 0.000000‐0.000102). This indicates that influence was concentrated among a small subset of users. Nodes with high degree centrality, such as U4839 (0.230 in 2022) and U65 (0.220 in 2017), served as major drivers of information propagation and significantly contributed to network vitality. Several nodes (eg, U1958 and U4813) maintained high degree centrality across multiple years, indicating their sustained impact on network activity and information spread (Figure 4A).

Nodes with high eigenvector centrality (eg, U1727) functioned as hubs for information flow, enhancing both global connectivity and transmission efficiency through connections with other central nodes (Figure 4B). Those with high betweenness centrality, such as U5091 (0.080 in 2023), played bridging roles across subgroups and were crucial for information exchange between different user communities. Node U4813 consistently exhibited high betweenness centrality over several years, confirming its ongoing role as a critical bridge (Figure 4C).

**Figure 5.**
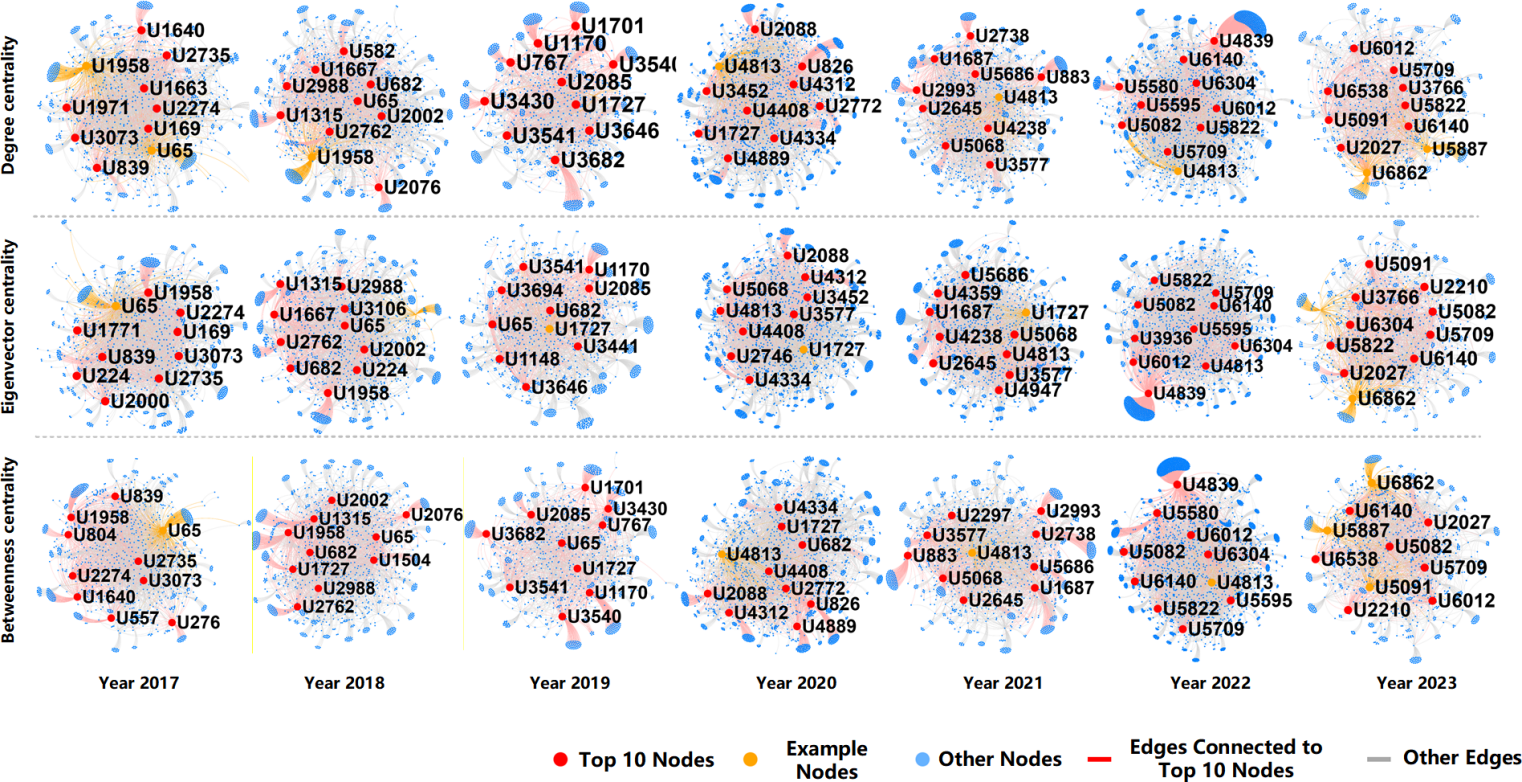
Evolution of core subnetworks.

Subnetwork analyses of core nodes further clarified their structural roles over time (Figure 5). For instance, in 2017, node U65 possessed the largest subnetwork with the most direct connections, indicating not only extensive influence but also a key role in promoting network activity and information dissemination. By 2023, nodes U6862 and U5887 showed increased subnetwork size and density, underscoring their importance in that year’s discussions. Notably, new core nodes emerged annually—similar to “super-users” or “influencers” roles—and rapidly gained influence through extensive interactions or connections with other high-weight nodes. These nodes considerably enhanced network activity and structural richness, serving as essential elements in maintaining platform stability and engagement.

To further link topic modeling with social network structure, we conducted a topic-network coupling analysis among posts and comments (Table 2). Feedback on living facilities had the largest discussion volume (1026 posts and 26,152 comments). However, this topic showed the lowest median centrality values among all categories (degree centrality: 0.000934, IQR 0.00052-0.002802; eigenvector centrality: 0.006551, IQR 0.002883-0.015684; and betweenness centrality: 6.84×10^−6^, IQR 0-0.000355). Feedback on campus services also had a large volume of discussion (863 posts and 11,580 comments) and showed moderate median centrality values (degree centrality: 0.001933, IQR 0.000846-0.005330; eigenvector centrality: 0.010230, IQR 0.003896-0.025705; betweenness centrality: 1.05×10^−4^, IQR 0-0.001447). In contrast, several lower-frequency topics were associated with higher network centrality. Current affairs included 195 posts and 791 comments but had the highest median degree centrality (0.003383, IQR 0.000846-0.010986) and eigenvector centrality (0.015800, IQR 0.004713-0.046867). Psychological and emotional support included 102 posts and 1510 comments and showed the highest median betweenness centrality (3.62×10^−4,^ IQR 0-0.005164). These findings suggest that topic prevalence and network position captured different dimensions of student online communication.

**Table 2.** Topic-level engagement and centrality measures of users.

Topic	Posts, n	Comments, n	Users, n	Degree centrality, median (IQR)	Eigenvector centrality, median (IQR)	Betweenness centrality, median (IQR)
Feedback on living facilities	1026	26,152	4969	0.000934 (0.00052-0.002802)	0.006551 (0.002883-0.015684)	6.84×10^–6^ (0-0.000355)
Information inquiry	1004	9628	2618	0.001712 (0.000745-0.005216)	0.009398 (0.003364-0.024412)	8.87×10^–5^ (0-0.001363)
Medical education	883	7185	2234	0.001817 (0.000745-0.005497)	0.008523 (0.002408-0.024874)	1.11×10^–4^ (0-0.001596)
Feedback on campus services	863	11,580	2660	0.001933 (0.000846-0.005330)	0.010230 (0.003896-0.025705)	1.05×10^–4^ (0-0.001447)
Campus policies	566	2096	879	0.002960 (0.000846-0.008879)	0.013412 (0.003778-0.039631)	2.57×10^–4^ (0-0.003304)
Current affairs	195	791	428	0.003383 (0.000846-0.010986)	0.015800 (0.004713-0.046867)	3.59×10^–4^ (0-0.004665)
Psychological and emotional support	102	1510	660	0.003197 (0.000934-0.010902)	0.013217 (0.003406-0.045674)	3.62×10^–4^ (0-0.005164)

## Discussion

### Principal Findings

This study integrated topic modeling, temporal topic analysis, and SNA to characterize the behavioral patterns, primary concerns, and dynamic community structures of medical students within the online BBS forum over 2017 to 2023. After consolidating semantically overlapping themes, 7 major topics were identified, led by feedback on living facilities, information inquiry, and medical education. The temporal topic analysis further showed that some concerns, particularly living facilities and campus services, remained persistent across years, whereas other topics, such as current affairs, showed more time-sensitive changes. Network analyses further revealed complementary diffusion roles of nodes with high degree, high eigenvector, and high betweenness centrality. Dynamic subnetwork analysis demonstrated both the dynamic turnover of core influencers across years and the persistent influence of a few key nodes, reflecting the generational evolution of the social community. The topic-network coupling analysis extended these findings by showing that topic prevalence and network position captured different dimensions of online student communication: high-volume topics were not necessarily network-central, while several lower-frequency topics involved users occupying more central or bridging positions.

The observed fluctuation in annual post counts, characterized by a nadir in 2019, a peak in 2022, and a subsequent decline, suggests that major public health events, notably the COVID-19 pandemic, influenced online engagement. During periods of stringent containment measures, the BBS appears to have served as a critical alternative channel for online communication, information seeking, and potentially emotional support, consistent with prior research showing that people increasingly rely on digital platforms for social connection and support during crises [25,26]. The subsequent decline after 2022 likely reflects a migration of interactions back to offline settings as campus life normalized. The stable activity window between 10 AM and 10 PM offers a practical opportunity for communication. This presents a concrete opportunity for educators and administrators to implement “time-sensitive” interventions. Important announcements, well-being check-ins, or prompts for feedback could be scheduled for posting during these hours to maximize visibility and engagement. Conversely, support services (eg, mental health resources and academic advising) could promote their availability during these peak times to better meet the needs of students.

The temporal topic analysis showed that, for students undergoing intensive medical training, everyday conditions such as dormitories, transportation, and campus services may shape their learning experience and sense of institutional support. At the same time, the increase in current affairs–related discussions in later years indicates that the BBS also functioned as a space where students responded to broader social, public health, and technological developments.

Topic modeling showed that living facilities and service-related topics accounted for the largest share of discussion, a finding that resonates with Maslow’s hierarchy of needs [27]. The prevalence of concerns about dormitories, canteens, libraries, and transportation suggests that for medical students operating under intense pressure, the fulfillment of basic physiological and safety needs serves as a foundational prerequisite for engaging in higher-level academic and professional pursuits. This finding corroborates the work of Verdone et al [28] and Tinto [29], which established a strong link between campus environment, student well-being, and sense of belonging. The forum functions as a vital, informal information commons, fulfilling needs not fully met by official channels. For administrators, the observed topic distribution provides an empirically grounded basis for prioritizing resource allocation and quality improvement.

This study applied SNA and text mining to the field of medical education research, providing an empirical view for understanding medical student group behavior. Our findings reveal a landscape of influential nodes within the student social network, where distinct types of key nodes fulfill specialized roles in information diffusion. This understanding of social network structure carries significant implications for peer support, intergroup communication, and policy dissemination. Nodes with high degree centrality are the most active “information drivers,” establishing broad connections through frequent interactions. Nodes with high eigenvector centrality represent individuals whose opinions carry significant weight and influence due to their association with other well-connected peers. In the context of medical education, these influential voices are pivotal for communicating validated emotional and academic support [30]. Furthermore, the results show that nodes with high betweenness centrality are indispensable, acting as crucial bridges connecting disparate subnetworks (eg, students from different academic years or specialized tracks). These results suggest a potential network-informed approach for understanding student communication and support needs in medical education. Such analyses may help administrators understand not only what students are discussing but also which users or groups are structurally positioned to facilitate information exchange. This transition from passive observation to strategic identification provides a credible and targeted pathway for implementing new policies, effectively disseminating critical health information, and optimizing campus services.

The topic-network coupling analysis further demonstrates that a frequently discussed topic was not necessarily a network-central topic. Feedback on living facilities generated the largest volume of discussion but showed the lowest median centrality values, suggesting that these concerns were broadly distributed across the community rather than concentrated among highly central users. This pattern indicates that high-prevalence topics may represent widespread everyday concerns that are collectively expressed by many users, even if they do not depend on core actors for visibility. In contrast, several lower-frequency topics, including current affairs and psychological and emotional support, were associated with higher centrality values. For administrators, high-frequency but dispersed topics may indicate broad-based needs requiring resource allocation or service improvement, whereas lower-frequency but network-central topics may indicate issues that circulate through influential or bridging users and may therefore require targeted support strategies. In practice, administrators could implement this approach through periodic evaluation of topic prevalence and network centrality rather than relying only on feedback or complaint volume. Degree centrality may help identify highly visible and actively discussed issues that require timely operational responses, such as service disruptions or frequently requested information. Eigenvector centrality may indicate topics circulating among well-connected users and can be useful for identifying issues that are likely to spread widely through the student community. Betweenness centrality may be particularly useful for detecting bridging topics or users that connect otherwise separated subgroups, suggesting where targeted communication or support may prevent concerns from remaining fragmented.

The dynamic evolution of the social network structure offers insights into the temporal dimensions and generational turnover inherent in the medical education pathway. As senior students transition to clinical internships, prepare for postgraduate examinations, or graduate, their time and energy allocation changes. Consequently, they are gradually replaced in central network positions by newly emerging influential nodes. This continuous turnover ensures the ongoing renewal of community culture and vitality. However, a small number of core nodes that maintain influence across multiple years likely represent consistent figures, such as dedicated educational administrators or student organization leaders. Their sustained presence is crucial for preserving the accuracy of critical information, ensuring the continuation of traditions, and maintaining community stability. These dynamic findings urge educational administrators to view the online community not just as a platform but as a genuine educational ecosystem.

Finally, the discussion of psychological and emotional support deserves careful interpretation [31]. Although this topic accounted for a relatively small proportion of discussion, it showed the highest median betweenness centrality in the topic-network coupling analysis. This finding suggests that when psychological and emotional support was discussed, users tended to occupy more bridging positions in the network. For medical education, this finding reinforces the importance of integrating accessible mental health resources and peer support mechanisms into the broader student support ecosystem.

### Limitations

Despite the insights provided by this study, there are several limitations. First, this study focused on textual posts and comments and did not analyze nontextual elements, including images, videos, and emojis. As these elements may convey additional meanings, excluding them may have led to the loss of contextual information. Future studies could incorporate multimodal analytic approaches, including optical character recognition and multimodal and generative AI tools, to extract and analyze text embedded in images or screenshots. Second, the findings are derived from one BBS platform, which may limit the generalizability of the results to other institutions or digital environments. In particular, the topics discussed, activity timing, and patterns of psychological and emotional support identified in this study should be interpreted in relation to the local medical curriculum, academic calendar, assessment schedule, clinical training pathway, and student support system. Therefore, the findings should be understood as reflecting the interaction between online student communication and a specific medical education context. Third, due to the anonymity of user data, we were unable to obtain demographic or educational background information, such as gender, year of study, or academic performance. Future work should aim to incorporate more comprehensive user information to better reveal differences in networking behaviors across various student populations.

### Conclusions

In this 7-year longitudinal study, we integrated topic modeling, temporal topic analysis, and SNA to provide a comprehensive view of the concerns and community dynamics of medical students within an online BBS forum. The results reveal that the concerns of medical students extend beyond academic matters to include living facilities and service-related topics. The temporal topic analysis showed that some concerns were persistent across years, particularly those related to the learning and living environment, whereas other topics showed more time-sensitive changes. This indicates that online forums can capture both stable features of student experience and emerging concerns shaped by broader educational, social, or public health contexts. The SNA uncovered a dynamic structure of influence characterized by distinct and complementary roles for key nodes—information drivers (high degree), influential hubs (high eigenvector), and community bridges (high betweenness)—which play complementary roles in information diffusion and peer support. The annual evolution of these core influencers reflects the natural turnover within the student community while underscoring the importance of consistent, long-term participants who help maintain community stability. The topic-network coupling analysis further showed that topic prevalence and network position captured different dimensions of student communication. High-volume topics, such as feedback on living facilities, were widely discussed but relatively dispersed, whereas several lower-frequency topics, such as current affairs and psychological and emotional support, involved users with more central or bridging network positions. These findings suggest that online forums are not merely casual platforms but constitute an organic component of the medical education ecosystem. For educational administrators and student support services, combining topic prevalence with network position may provide a more nuanced approach to identifying student needs. High-frequency topics may indicate broad-based issues requiring resource allocation or service improvement, while lower-frequency but network-central topics may require targeted communication. Overall, this study provides a data-driven perspective for building more responsive and supportive medical education environments.

## Abbreviations

BBS: bulletin board system
LDA: latent Dirichlet allocation
SNA: social network analysis
